# Hospital and Institutional News

**Published:** 1914-11-14

**Authors:** 


### HOSPITAL AND INSTITUTIONAL NEWS.

### BOOTS FOR THE BELGIAN WOUNDED.

With reference to the numbers of pairs of boots required promptly to equip the wounded Belgian soldiers sent to England for treatment, referred to last week in these columns, we shall welcome full particulars of the need that exists from the authorities of each hospital which has Belgian soldiers under treatment. We therefore formally request every hospital where any such need has arisen to send to the Editor at once information of the number of Belgian wounded admitted as patients and the number of those who are in need of boots. We understand that each Belgian soldier will know his own size in boots, which should be ascertained and sent to us with the other particulars. We shall then be in a position, thanks to the co-operation of Messrs. Manfield and Sons, of Northampton, who are supplying the French Navy with boots, to arrange the necessary details for the dispatch of the right-sized boots as required. To succeed, the scheme must commend itself to our readers, and they must send us their names and subscriptions without delay. A subscription of 15s. 6d. will provide one pair of reliable and serviceable boots specially suited for the use of soldiers. We estimate that the money in hand, including the proceeds of a display of Church Scouts at Bath on the 19th instant through the Rev. B. Rees, will provide at least fifty pairs of boots. We shall be glad if all those of our readers who would like to co-operate will communicate with the Editor immediately, as this practical assistance to the Belgian soldiers, if it is to be effective, must be given promptly. It rests, therefore, with the readers of The Hospital to decide whether or not this simple method of being of practical help and comfort to a considerable number of wounded Belgian soldiers shall be made effective or shall be dropped. All letters on this subject should be addressed to the Editor, The Hospital, 28 and 29 Southampton Street, Strand, London, W.C., and the words "Boots for Belgians" written in the left-hand corner of the envelope.

### THE NORTH MIDLAND CLEARING HOSPITAL LEAVES FOR THE FRONT.

This unit, organised for service with the Expeditionary Forces, consisting of eight officers and seventy-seven men, left Leicester on November 5 for Luton, prior to being dispatched to the Front for active service. The commanding officer is Lieut.-Col. Pemberton Peake, and the following medical staff accompanies him: Lieuts. Costobadie and McNeill and Lieut. and Quartermaster Stokes, Leicester; Lieuts. Dixon, Melton Mowbray; Thomas, Market Harborough; Hood, Cresswell; and Andrew, Buxton. Prior to leaving the non-commissioned officers and men were presented by the Mayoress with suitable gifts of underclothing and parcels containing a flannel shirt, woollen socks, gloves, body-belt, a Balaclava helmet, and muffler, collected or made by Mrs. Pemberton Peake and Mrs. A. I. Groves. The unit took with it twenty-five tons of hospital stores.

### THE BOOT DIFFICULTY AT GRAVESEND.

Thirty of the wounded Belgian soldiers who were admitted into the Gravesend Hospital a fortnight ago have sufficiently recovered to report at headquarters in London. As many of the men when admitted into the hospital were without footgear, it was rather a difficult problem to find the necessary boots to enable them to report themselves. However, through the kind assistance of friends sufficient second-hand boots and shoes were forthcoming to enable the men to be decently shod, until such times as they could be refitted with clothes and equipment. In view of others in a similar plight when ready to leave the hospital, an appeal has been made through the local Press for cast-off boots or shoes having a little life still left in them to be sent to the hospital for the use of the heroic Belgians when they leave to convalesce. Another convoy of wounded filled all available beds on October 27. These men had taken part in the hard fighting on the Yser and around Dixmude, some not having had their boots off for seventeen days.

### THE SALARY OF AN IRISH HOSPITAL SECRETARY.

A very remarkable discussion on the salaries paid to its officers took place at the last monthly meeting of the committee of the County Kerry Infirmary. Mr. T. Harty moved that the salary of the secretary, Mr. W. Barrett, be increased from its present sum of £60 to £104 a year. They would not get a man at his present salary to wash bottles, and he had reduced the debit balance from £173 to £47 in the past half-year, had saved them £15 by doing away with board wages, and was a very good officer. In seconding the motion, Mr. R. D. Murphy declared that Mr. Barrett was the only secretary who had ever done his duty. In the course of further discussion Mr. Bernard said that 16s. a week at the time the secretary was appointed would be better than a pound a week now, and another speaker pointed out that the secretary was a whole-time officer. The Chairman, Mr. St. J. Donovan, said that he favoured the increase, but a rise of 80 per cent. after two and a half years looked a little big. If they increased the salary by increments it would look better. Mr. Murphy then remarked that he knew of no institution where officials were so badly paid as in the County Infirmary. He thought that the medical staff were entirely underpaid. After it had been unanimously decided to increase the salary, the Secretary, in returning thanks, said that it would not be his fault if there were not a credit balance next year. It is noteworthy that the previous secretary, Mr. James Coyle, used always to be there when wanted, and was virtually a whole-time officer, as the Chairman said; but it is surprising that any successor was found at such a small salary. The institution contains thirty beds.

### CARDIFF SCHEME FOR VISITS TO WOUNDED.

A movement to facilitate visits to wounded soldiers in hospital by wives and other near relatives has been started at Cardiff. Under the War Office Regulations a free railway ticket is issued only to the wife or relative selected by a wounded soldier who is "dangerously ill." The warrants for such facilities are granted subject to the discretion of the surgeon in charge, and only in cases where such relatives have no means of their own for travelling expenses. Under the scheme now contemplated, it is proposed to set up a small committee, nominated by the Lord Mayor, which shall collect funds to provide, where necessary, for the maintenance at Cardiff for three or four days in normal cases of the wife or relative visiting a wounded soldier under the War Office Regulations referred to. It is also proposed to extend this facility to the wife or relative of a wounded soldier who is *not* dangerously ill where the surgeon in charge certifies the case as one in which the wish of the soldier should be granted, and where the wife or relative has no means for travelling and maintenance expenses. The clergy are interesting themselves in the matter, and already have arranged for free board and lodging for a number of these cases at the houses of members of their churches. Colonel Hepburn, V.D., the officer commanding the 3rd Western General Hospital, approves of these proposals, and has provided office accommodation for the secretary of the committee at the hospital's headquarters, Howard Gardens, and will furnish as often as needs require a list of cases recommended for such visits.

### ACCOMMODATION FOR MENTAL DEFECTIVES.

Since the passing of the Mental Deficiency Act a great deal of extra work has been thrown upon the London Mental Hospitals Committee, in the way of providing accommodation for those defectives who require treatment. So far, tentative arrangements have been made with a few institutions to receive a limited number of defectives, but the committee feel that the County Council itself should make some provision, rather than continue to depend solely upon what can be obtained vicariously. In this accommodation separate provision will have to be made for various kinds of defect which will call for special classification. Among the cases which seem to the committee to call most urgently for provision are those of feeble-minded girls and young women, and they have been endeavouring to find premises which might be adapted without very much cost or the loss of much time for the use of a certain number of cases of this kind. Particulars of various properties have been considered, and it is proposed to purchase the buildings hitherto used as mission schools for Hebrew children at South Side, Streatham Common. The site of the property is some 27,000 feet super., with a frontage to Streatham Common of 160 feet. The freehold may be bought outright for £3,250. The scheme, tentatively approved, provides for the accommodation of about seventy defectives, with a staff of eight or ten.

### THE SALARY OF MENTAL HOSPITAL SUPERINTENDENTS.

Some interesting points were made at the recent quarterly meeting of the committee of visitors to the North Wales Counties Mental Hospital, Denbigh, with reference to the salary of the medical superintendent, Dr. Frank Jones. The house, staff, and supply committee recommended that his salary be increased by annual increments of £50, instead of £25, up to a maximum of £700. The post was advertised as being vacant at the end of Dr. Stanley Hughes' term at £500, rising to £700, and, as Mr. Gamlin remarked, it was felt that among the candidates Dr. Frank Jones' claim, based on many years' service, was the strongest. In spite of the resignation of one of their members, the speaker proceeded, Dr. Jones' appointment had been fully justified. His promotion had led to his salary being virtually doubled, but in June or July 1913 the question of accelerating his increment was discussed, after he had been about a year in his new post. The question then arose as to the reason why the staff and supply committee recommended the acceleration, which he understood not to have been put forward by the medical superintendent himself. Mr. J. R. Hughes, chairman of the staff and supply committee, then explained that the visitors favoured the increase, and called attention to a letter from Dr. Jones asking for an increase. He showed in this letter that his salary was the lowest in any institution of the kind, and, proceeded Mr. Hughes, after full consideration the committee came to the conclusion that the medical superintendent was much underpaid, although he had justified every expectation. He therefore proposed that the committee's recommendation should be adopted, which was unanimously agreed to. The result is interesting for various reasons. One is the unfortunate tendency that exists for a committee which promotes a man to a senior post to consider that such an officer is, as it were, indebted especially to them. The truth is that each man must be judged on his own merits, and once he has been appointed, no matter from within or without the institution, he has a right to insist that the standard prevailing generally in the position which he occupies should be reflected in his salary, quite apart from his having been promoted or not.

### THE CELEBRATION OF A JUBILEE.

A deputation of three employees of the Co-operative Wholesale Society, Ltd., Boot and Shoe Works, at Leicester and Enderby, attended a recent meeting of the committee of the Leicester Royal Infirmary to present to the Children's Hospital a piano which had been contributed for by the employees in commemoration of the jubilee of the Society. The gift was accepted by Mr. Fielding Johnson, the chairman, and Mr. C. J. Bond, who both expressed deep appreciation of the motive which had prompted such an acceptable gift. It is all to the good of the voluntary hospitals that bodies of workpeople should take a lively interest in all that concerns the happiness of patients, and we have always held the opinion that many needs might be met by valuable gifts if only the workers could be brought into such close touch with institutions that the actual needs could be really appreciated by them at first hand.

### MEDICAL MEN AS PRISONERS OF WAR.

In view of the leading article which we published on this subject in our last issue, it is interesting to read the following passage from the statement which has been issued by the Foreign Office with reference to the exchange of British subjects in Austria-Hungary and Germany for Austro-Hungarian and German subjects in Great Britain and Ireland: "Arrangements have been made," the statement proceeds, "with the Austro-Hungarian Government whereby women and children, male British subjects under eighteen and over fifty years of age, together with doctors, ministers of religion, and invalids even within these age limits, are allowed to return from Austria or Hungary in return for reciprocal treatment here. Inquiries are being made as to the number of Austro-Hungarian subjects in the British Isles of military age who have not undergone military service, and when these are completed proposals will be made for the exchange of these persons for the same number of British subjects of a similar nature who are now detained in Austria-Hungary." In regard, moreover, to British subjects in Germany, the statement adds: "An agreement has also been made permitting the reciprocal return of the male subjects of both countries under seventeen and over fifty-five, and of doctors and ministers of religion. In spite of this agreement, which was completed on October 22, four very elderly invalid retired officers, two clergymen, and a doctor are still being detained at Bad Nauheim or Frankfurt, and several protests against their detention have been made through the American Embassies in London and Berlin. Proposals were made a month ago for the exchange of invalids by the British Government, and of persons who, owing to weakness or physical disability, were not likely to make useful soldiers, as well as of all persons who had not undergone military training. These proposals have been refused." Thus at all events a beginning has been made in the restitution of medical men by all sides to the countries to which they belong. Is it too much to hope that arrangements may also be concluded for the prompt exchange of medical prisoners of war?

### THE LEAVEN OF PROGRESS.

We are glad to see the quiet progress that is being made with the installation of up-to-date $x$-ray apparatus throughout the Poor-Law infirmaries. The latest instance is that of Wrexham Infirmary, where the Board of Guardians have conceded the request of Dr. Moss, the medical officer, and decided to provide the infirmary with an installation at a cost of £250. As an instance of how one useful piece of work leads to another we may note that this institution is now accommodating 100 Belgian wounded, and no doubt their need for this aid to diagnosis much influenced the Guardians' wise decision. Thus when peace is at last restored a step forward will have been gained, and the general standard of equipment permanently raised, with the result that the $x$-ray apparatus will become permanently useful in treating Poor-Law cases when the infirm are once more in full possession of the building. Indeed, it cannot be emphasised too much how valuable is any one worthy addition to an institution. Each new piece of well-considered equipment acts like leaven, creates new needs, and, finally, succeeds in bringing the whole standard of its department up to its own level.

### HOSPITAL CONCERTS FOR LONDON RECRUITS.

The Board of Management of the London Temperance Hospital, Hampstead Road, have given permission for the out-patient department to be used for concerts to the recruits and Regulars in the district. The concerts, which are organised by the hospital staff, are to be held every Saturday until further notice. As the headquarters of the 3rd Battalion County of London Royal Fusiliers are situated quite near to the hospital, this action of the staff is much appreciated by the officers and men. It will be remembered that the out-patient department of the London Temperance Hospital is quite distinct from the main hospital building, so that no discomfort is caused to the patients. We have previously reported the fact of out-patient rooms being used by the military authorities as recruiting stations, and now we add an instance of an out-patient department as a concert-hall for the military.

### GLASGOW HOSPITALS AND MUNICIPAL CONTROL.

The Glasgow Town Council is the first municipality to adopt our suggestion and appoint a special committee to inquire into and report upon the adequacy for the city's requirements of the facilities for the institutional treatment of disease. The scope of the inquiry is to cover the public institutions as well as those supported by voluntary contributions, and is directed with a view to ascertaining whether or not any augmentation is necessary. The committee are also asked to consider and report as to the desirability of co-ordinating all the institutions covered with a view, we hope, to their being placed under an Advisory Council on which each hospital and also the corporation will be adequately represented. Municipal ownership is already in an advanced stage in Glasgow, and the possibility of the whole of the public medical services of the city being directly absorbed by the civic administration is not practicable, but the adoption of our suggestion points the way to progress.

### POOR-LAW INFIRMARIES AND THE WAR.

At a recent meeting of the Edmonton Board of Guardians a letter was read from the General Officer Commanding-in-Chief, Eastern Command, asking whether the Guardians' offer of beds holds good for the sick of the new armies and local troops as well as for the sick and wounded of the Expeditionary Force. It was agreed that the authorities be informed that the offer of beds holds good for the sick of the new armies and local troops. The Guardians had also been asked if they agreed to the rate of pay offered—the same as for general hospitals—based on a payment per day for each bed occupied. It was agreed that this was satisfactory. Much added work has been thrown on the infirmary staff, due to the treatment of sick Belgian refugees from the Alexandra Palace, especially in the maternity wards of the institution. Incidentally, we may note that Edmonton, in common with other Poor-Law authorities, has had no applications for the post of assistant medical officer, and the board have decided to re-advertise the post at an enhanced salary.

### RADIUM IN POOR-LAW INFIRMARIES.

The Wandsworth Board of Guardians have received a report from Dr. W. L. Maccormac, medical superintendent of St. James's Infirmary, with reference to the use of radium in the infirmary, which was referred to in the columns of The Hospital on October 17, page 59. Dr. Maccormac points out that the price of radium still remains at £20 per milligramme, and he was of opinion that at least 50 milligrammes would be necessary in the infirmary for satisfactory work. The Radium Institute had in its possession 4,000 milligrammes of radium, valued at £80,000, and the chairman of the committee, Sir Frederick Treves, thought that was more than all the radium in the world used outside the Institute. A large proportion of that sold was of half-strength or less. Judging from recent literature, Dr. Maccormac adds, the opinion obtained was that, even bearing in mind the nature of the cases treated, the results were frequently disappointing. Because of the inoperable nature of the cases treated it would be unwise to compare the results obtained with the statistics of ordinary surgery. But, in contrast with the usual treatment of alleviation, or policy of unrestraint, the results were undoubtedly gratifying. In conclusion, Dr. Maccormac said that he would like to see his infirmary possess radium, but in view of its cost and scarcity he did not feel justified in pressing for a supply.

### SPECIALISTS FOR LONDON INFIRMARIES.

In the past he had obtained admission for selected cases to the Cancer Hospital, but it was impracticable to arrange for the admission of anything but a fraction of the number of cases in the wards who would, in his opinion, stand to benefit from the use of radium. The Infirmary Committee, in view of this report, did not recommend the board to take any action, but Mr. D. Grundy, who has taken a prominent part in pressing it on the board, proposed that the Guardians apply to the Local Government Board for permission to purchase radium to the value of £1,000. Mr. P. Rees suggested that it would be better to approach the Metropolitan Asylums Board, and inquire whether that authority was prepared to provide facilities for the treatment of cancer cases by radium. Half a dozen specialists, he added, could give the treatment in the whole of the infirmaries of London. The board rejected the proposal made by Mr. Grundy, and decided to leave the matter with the medical superintendent.

### NO FUNDS FOR SANATORIUM BENEFIT.

A serious position was revealed at a meeting last week of the Walsall Insurance Committee, presided over by Dr. J. J. Lynch, with regard to the inadequacy of the funds for sanatorium benefit at the committee's disposal. The estimated income for the year was given as £1,423, of which £1,268 had been already spent, so that only £155 was left, which would probably mean an overdraft at the end of the year of £300. Dr. Lynch declared that the only way out of the difficulty was to put in operation the section of the Act which provided that any such excess should be paid by the Treasury and the Town Council in equal proportions. He therefore moved that the Commissioners be approached, since there were many people in the town who were expecting sanatorium treatment, without which their health might be sacrificed. Certainly the present is no time for withholding any form of institutional treatment offered by the Act, and the results, serious enough in the past, would be doubly serious now in the present crisis of war and with the approach of winter.

### PRISON DOCTORS AND ACTIVE SERVICE.

All the public services have provided their quota of volunteers to the armed forces of the Crown, and the Crown civilian services themselves have not been found wanting. Thus we find that some four hundred officers from H.M. Prison Service have been enrolled in the Army or Navy. These include a large number of hospital officers who are in the R.A.M.C. or other corps. Dr. S. S. H. Shannon, deputy medical officer, Wakefield Prison, is now staff-surgeon on board H.M.S. *Hermione*, and Dr. A. W. French, deputy medical officer, Portland Prison, is serving as captain in the R.A.M.C.(T.).

### THE INFLUENCE OF RELIGION ON MENTAL PATIENTS.

In the latest annual report of the County Borough of Ipswich Mental Hospital, of which Dr. Edmund L. Rowe is medical superintendent, an interesting passage occurs with reference to the value of religious ministrations in exercising a beneficial influence on the patients. Mr. J. A. C. V. de Candole, the hospital chaplain, first alludes to the refurnishing of the chapel, and to the æsthetic and practical improvement resulting from it. But he does not stop there. "It cannot be said," he adds, in spite of encouragements, "that the ministrations of religion have yet been at all adequately tested as to their power of exercising a beneficial influence on the patients. Much remains to be done." Mr. de Candole does not particularise, though he suggests that further classification of patients may provide greater opportunities. We ourselves have often noted the institutional value of properly conducted services in hospital chapels, and not long ago quoted a medical superintendent as remarking that such services lost much of their value to the staff when performed in rooms generally devoted to lay use. Practical experience, then, supports Mr. de Candole's attitude, and it would be interesting to learn how he would propose to test its usefulness further, and what means he would advocate for this end.

### A GUERNSEY HOSPITAL PRESIDENT RESIGNS.

The Board and the Country Hospital of Guernsey have sustained a loss in the resignation of Mr. Thomas Robin from the office of President. He has held the position for a period of ten years, and during this time many alterations have been carried out. The number of beds in the institution at the present time is 150.

### A CITY HOSPITAL FOR THE WOUNDED.

Generosity and Mansion House Funds are always associated in our minds with the City, and we are apt even to forget the ambulance work by which it has long set an example. As a reminder, therefore, of its institutional and first-aid proclivities comes the announcement that the City Red Cross authorities are about to provide a hospital in the City for the wounded. Indeed, a beginning was made when Fishmongers' Hall was first turned into a hospital for officers. If the scheme successfully matures it will be only the latest example of the active part taken by the City in every phase of the nation's life.

### A WEAK POINT IN THE "FOLLOWING-UP" SYSTEM.

"It is in following up tuberculous cases and contacts" that the strength of prevention lies, and any means by which it is ensured is well worth many times the cost of a few notification fees. A weak point in the chain is the fact that the transference of a patient from one institution for tuberculosis to another is not notified. By such breaks in continuity following up is hampered, patients are lost sight of, and the preventive value of notification is reduced. Here is something small, necessary and practically possible for the anti-phthisis campaign to achieve. It would be worth all the so-called health weeks in the United Kingdom.

### FINAL CONVALESCENT HOME ARRANGEMENTS IN SCOTLAND.

The question of the control of convalescent and hospital accommodation in Scotland has been subject to more than one alteration since the war began, but as the latest arrangement appears final it is well to note its main provisions. The executive of the Scottish Branch of the British Red Cross Society has received instructions from the War Office to the effect that all arrangements for making use of convalescent home and hospital accommodation in Scotland have been placed in the hands of the Red Cross Commissioners of the four Scottish districts, to which allusion has been made already in The Hospital. Consequently all offers of such accommodation which have not yet been made use of should be communicated to the Commissioner of the district in which the home or building is situated. The names of the four Commissioners are: Western District, Colonel R. D. McEwan, headquarters, Glasgow; Eastern District, Mr. David Wallace, C.M.G., headquarters, Edinburgh; North-Eastern District, Colonel Scott-Riddell, M.V.O., headquarters, Aberdeen; Central Eastern District, Colonel Gordon Thomson, V.D., headquarters, Dundee.

### THE NEW INTERNATIONAL HOSPITAL AT TOKYO.

Count Okuma has announced the gift of £10,000 from the Emperor of Japan towards the foundation of St. Luke's International Hospital at Tokyo, an undertaking which has been largely promoted by the Episcopal Mission. The movement in favour of the new institution has received substantial support in America, and according to Reuter's correspondent the hospital itself will be the most elaborately equipped institution of its kind in the Far East. The scheme, which is expected to cost £200,000, and is, of course, supported by both Americans and Japanese, is also of interest to voluntary hospital workers from the fact that the Emperor by its means is understood to wish to solidify international understandings. How significant a fact is this for ourselves in England, the cradle of the voluntary system, where we have all been taught how much solidarity and good have accrued from the practice of that gospel of personal service in the days of health for the cause of the sick, which is what our own institutions stand for! Since the voluntary hospital in this country has, as a matter of historical fact, permeated even the old Poor-Law institutions with its spirit, and bridged all classes by its answer to the appeal of sickness, may we not hope that it will also serve to promote understandings in the larger sphere which the Emperor of Japan has chosen now for its province? St. Luke's International Hospital at Tokyo being, in fact, a Japanese-American hospital, will, we hope, do something to check the national rivalries between these two races, which have threatened more than once in the past to collide.

### THE GATE OF ENGLISH HOSPITALITY.

The hospital with which perhaps most sympathy will be felt by administrators at the present time is the Royal Victoria Hospital at Folkestone. As we all know, Folkestone has been the Gate of England for refugees from Belgium, and an amount of personal service has been required from the inhabitants of that resort of late to which they have well responded and for which they themselves must be all the better. But, apart from refugees, many wounded Belgian soldiers have also arrived, with the result that the resources of the institution have been strained to the uttermost. The chairman, Mr. Stephen Penfold, and the senior physician, Dr. W. J. Tyson, now complain not merely of a debt of £1,350, but also of "a rapidly decreasing surgical stock," to say nothing of "a lack of extra comforts." The new arrivals—the new batches of wounded—are a more noteworthy feature at Folkestone than elsewhere on account of its situation; but this fact ought to have a stimulating effect upon subscribers. If these only realised it, they have what is in one sense a unique opportunity for raising a memorial to those fallen in the war by providing every facility for the extension of the hospital's work. Last week no fewer than 108 Belgian soldiers were under the hospital's care; and if the claims are further loyally met Folkestone will be in the proud position of having shouldered the double burden of refugees and wounded, and made the Gate of England a gate of hospitality such as no other town can show. Mr. J. Kennett, the secretary, has surely here the material for focussing public enthusiasm that only comes in a hospital manager's way once in a generation.

### DEATH OF BEAU NASH'S SUCCESSOR AT BATH

The Bath Royal United Hospital by the death of Major Simpson has lost another of its valued trustees. Major Simpson, who occupied the unique position of Master of Ceremonies of Bath, was, like his historic predecessor, Beau Nash—as recorded in The Hospital of March 7, 1914, p. 627—keenly alive to the claims of hospital work, and it was largely owing to his influence in opening and supporting a special fund during his second mayoralty that the financial crisis of the Royal United Hospital was satisfactorily met. The late M.C. had on one occasion the honour of being entertained by the local medical profession, and was elected on the first board of management of the Winsley Sanatorium.

### THE "WAR SCHEDULE" OF INSURANCE DRUGS.

It will be remembered that a few weeks after the commencement of the war the Insurance Commissioners, recognising that panel chemists would be bound to incur heavy losses if required to dispense at tariff prices drugs which had seriously increased in cost as a result of the war, agreed to sanction the payment of special prices for certain drugs. These drugs were comprised in a list called the "War Schedule," but they numbered of the drugs whose prices had risen as a direct consequence of the war. The Commissioners have now agreed to the addition of ten other drugs to the list, which now consists of the following substances: Thymol, acetyl-salicylic acid, salicylic acid, sodium salicylate, dilute hydrobromic acid, vinegar of cantharides, ammonium bromide, potassium bromide, sodium bromide, ichthyol, bismuth carbonate, bismuth oxide, bismuth salicylate, bismuth subgallate, bismuth subnitrate, chloral hydrate, cantharides plaster, solution of bismuth and ammonium citrate, malourea, phenacetin, phenazone, potassium permanganate, salicin, sodium theobromine salicylate, and paraldehyde. Payment will be allowed for these drugs on the basis of current wholesale prices. The list of drugs which have advanced in price as a result of the war is much longer than that comprised in the "War Schedule" even now it has been extended.

### THE LONDON AMBULANCE SERVICE.

It is good news to learn that the motor ambulances ordered by the London County Council will shortly be delivered and ready for service. Owing to the war, the field from which skilled motor drivers and ambulance attendants can be drawn is limited, and it is therefore proposed that during the war temporary employees shall be engaged, and the appointment of permanent staff deferred until the termination of the war. At each of the five stations where one ambulance is provided it is proposed to provide a staff of seven attendants, working in two twelve-hour shifts of three men each, with an extra man as relief. At the station with two ambulances the staff would consist of fourteen men. The men will receive 35s. a week, but the head attendant at each station will receive five shillings more. Four telephone operators are also to be appointed for duty at the central station. The estimated cost of the staff when the scheme is in full working operation is £5,075 a year. The County Council has already authorised the appointment of a principal assistant, at a salary of £400 to £500 a year, to supervise the service, but it is not proposed to make this appointment at present. A temporary assistant is to be engaged, however, at a salary not exceeding £10 a week.

### THE ART OF APPEALING.

In The Hospital of July 4 last, on the first page of the *Institutional Worker*, we reproduced an appeal showing brains and originality. That appeal was concentrated, pointed, diagrammatic, and effective, because it was well thought out and skilfully designed. It may have, and we hope has inspired others to appeal successfully on similar lines. One such attempt has been sent us, however, which graphically illustrates why appeals fail. This appeal introduces the diagrams but blankets them out by ill-chosen type and surplus words, which are fatal to its success. The truth is that all workers, whether for hospitals or kindred institutions, must be trained in every department of the work, or they cannot fail to give evidence of having mistaken their vocation; since only by such training can they improve the institution with which they may become associated.

### THIS WEEK'S DRUG MARKET.

The continued arrival of drugs from neutral ports has the effect of preventing any general advance in prices, but the supplies of some products, notably synthetic drugs, are slowly diminishing. Considering the circumstances, the tone of business is very fair, and there has been some improvement in the demand. The cutting off of supplies from Turkey must inevitably cause an advance in the prices of opium, morphine, and codeine. Salts of mercury are again dearer, and carbolic acid continues to tend upwards in price. It seems probable that the high values of mercurials and carbolic acid will be maintained. Citric acid, tartaric acid, and cream of tartar continue to tend downwards in price, and there has been a slight reduction in the quotation for iron and quinine citrate. Otherwise fluctuations in value have been few and unimportant.

